# The Eurasian Magpie Preys on the Nests of Vinous‐throated Parrotbills in Invasive Smooth Cordgrass

**DOI:** 10.1002/ece3.70905

**Published:** 2025-01-23

**Authors:** Yanhong Chen, Youle Xu, Junjie Wang, Taiyu Chen, Bin Liu, Pan Chen, Changhu Lu

**Affiliations:** ^1^ College of Life Sciences Anhui Normal University Wuhu China; ^2^ College of Life Sciences Nanjing Forestry University Nanjing China; ^3^ Management Bureau of Dafeng Milu National Nature Reserve Yancheng China

**Keywords:** biological invasion, Nest predation, *Pica pica*, *Sinosuthora webbiana*, *Spartina alterniflora*

## Abstract

Native animals worldwide are experiencing long‐term coexistence with invasive plants, leading to diverse behavioral changes. Invasive plants may create new habitat structures that affect the distribution or behavior of prey, which in turn might attract predators to these novel habitats, thereby altering predator–prey dynamics within the ecosystem. However, this phenomenon is rarely reported. Our previous research found that in the Yellow Sea wetlands of China, the native bird species, the vinous‐throated parrotbill ( *Sinosuthora webbiana* ), has adapted to breeding in the invasive smooth cordgrass ( *Spartina alterniflora* ) by increasing its nesting height. Here, our observations indicate that in cordgrass habitats, the main nest predator of parrotbills was the Eurasian magpie ( *Pica pica* ), accounting for 75% of predation events. In contrast, in native habitats, the primary predators were mammals and snakes, accounting for 83% of predation events, with no nests being predated by magpies. We believe that changes in the breeding and nesting behavior of parrotbills may have attracted magpie predation in cordgrass habitats. Our findings may provide an empirical case of how behavioral changes induced by invasive plants can lead to dynamic shifts in predation relationships. We advocate for further research into this intriguing phenomenon, as it could enhance our understanding of changes in interspecific relationships and their ecological consequences within the context of biological invasions.

## Graphical abstract

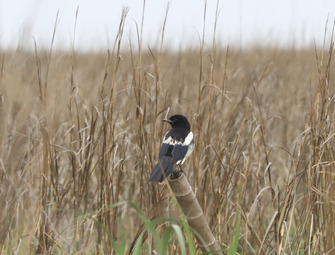

## 1 Introduction

Biological invasions refer to the introduction and establishment of non‐native species in new ecosystems, occurring in nearly every region of the world, and are recognized as one of the leading threats to biodiversity (Butchart et al. 2010; Crystal‐Ornelas and Lockwood 2020). Invasive plants are among the most widespread invasive taxa, frequently causing significant changes to both the biotic and abiotic characteristics of ecosystems (Davidsdottir et al. 2016). Worldwide, native animals are experiencing long‐term coexistence with invasive plants and are obliged to adapt to the reshaping of habitats by these plants, resulting in diverse behavioral variations (Stewart et al. 2021). This behavior change mediated by invasive species may spark an ecological chain reaction, affecting predator–prey dynamics (Kamaru et al. 2024).

Nest predation is an important limiting factor for the reproductive success of birds, affecting their life cycle and population dynamics (Ibanez‐Alamo et al. 2015; Weatherhead and Blouin‐Demers 2004). The composition of predators may change depending on habitat characteristics and the identities, behaviors, and activity patterns of both predators and prey (Cockle et al. 2016; Cox, Thompson III, and Faaborg 2012; Thompson and Burhans 2003). In wetland ecosystems, hydrological conditions have a significant impact on birds' nest site selection, nest predation rates, and the composition of nest predators (Gjerdrum, Elphick, and Rubega 2005; van Oort et al. 2015; Robertson and Olsen 2015). The rise in tide or river water levels increases the risk of bird nests being submerged (Yilmaz et al. 2020). However, some bird species are adaptable and adjust their nesting timing, height, and location to mitigate this risk (Gjerdrum, Elphick, and Rubega 2005; Clauser and McRae 2016). Differences in nest survival rates may also be attributed to variations in predator communities (Twedt et al. 2001), with changes in wetland water levels potentially altering predator activity patterns and distribution (van Oort et al. 2015). Rising water levels may submerge terrestrial predator habitats, reducing their threat to bird nests. Conversely, falling water levels might expose more predators, such as mammals or reptiles (Schmidt et al. 2023). Therefore, the reduction in nest predation during flood conditions could offset the increased risk of nest inundation (van Oort et al. 2015). When faced with the trade‐off between predation and tidal inundation, seaside sparrows ( *Ammodramus maritimus* ) prioritize nesting in lower areas to avoid predation risk, but they will move to higher areas for nesting after experiencing tidal flooding (Hunter, Nibbelink, and Cooper 2016).

Invasive plants can sometimes create new habitat types for native birds by offering less competitive blank ecological niches, thereby attracting birds to these areas (Stinson and Pejchar 2018). Some studies have shown that habitat landscape changes caused by invasive plants may alter the composition of predators and nest predation rates (Cook and Toft 2005; Fisher and Davis 2011). For instance, the specific vegetation architecture of invasive plants can enhance or reduce nest concealment (Grant et al. 2006; Schmidt and Whelan 1999; Smith, Finch, and Hawksworth 2009), and modify predator habitat selection, foraging effort, and efficiency (Borgmann and Rodewald 2004; Rodewald, Shustack, and Hitchcock 2010; Schlossberg and King 2010). The types of native predators and invasive plants may jointly affect nest predation relationships (Lyons et al. 2015), but there are few reports on predator–prey dynamics, especially in wetland ecosystems.

Smooth cordgrass ( *Spartina alterniflora* ), native to the Atlantic coast of North America, was introduced to China in the 1970s for the purpose of promoting siltation and embankment protection (Gao et al. 2012; Zuo et al. 2012). However, its distribution has rapidly expanded over the past few decades, profoundly impacting China's coastal landscapes, biological communities, and ecosystem functions (Li et al. 2009; Ning et al. 2021). Early studies revealed that most native birds avoid pure cordgrass habitats due to their high stem density and monoculture (Gan et al. 2010), but as these native birds and pure cordgrass coexist over time, some birds gradually move into invasive cordgrass habitats (Chen et al. 2022, 2023; Ma et al. 2014; Wu et al. 2023).

In the Yellow Sea wetland of China, our previous study revealed changes in the nesting behavior of a native bird, the vinous‐throated parrotbill ( *Sinosuthora webbiana* ), in long‐term invasion habitats of cordgrass (Chen et al. 2023). In native habitats, this small shrub‐dwelling bird (with a body length of about 11–13 cm) typically nests in the lower parts of native vegetation, possibly to increase nest concealment and avoid intense nest site competition with other sympatric species. However, in invasive cordgrass habitats, their nesting height increases, with nests positioned in the middle to upper parts of cordgrass, which helps parrotbills withstand periodic flooding risks (Chen et al. 2022). Changes in adaptive behavior may modify species interactions through trophic pathways (Kamaru et al. 2024). We predict that native predators will follow parrotbills into invasive cordgrass habitats and that changes in parrotbill nesting behavior may lead to shifts in the composition of potential nest predators.

To understand the cascading effects of nesting behavioral variation on the identity of nest predators, we monitored nests of parrotbills in native vegetation and invasive cordgrass habitats during a complete breeding season, and identified nest predators at each habitat.

## 2 Methods

### 2.1 Study Site and Subjects

**FIGURE 1:**
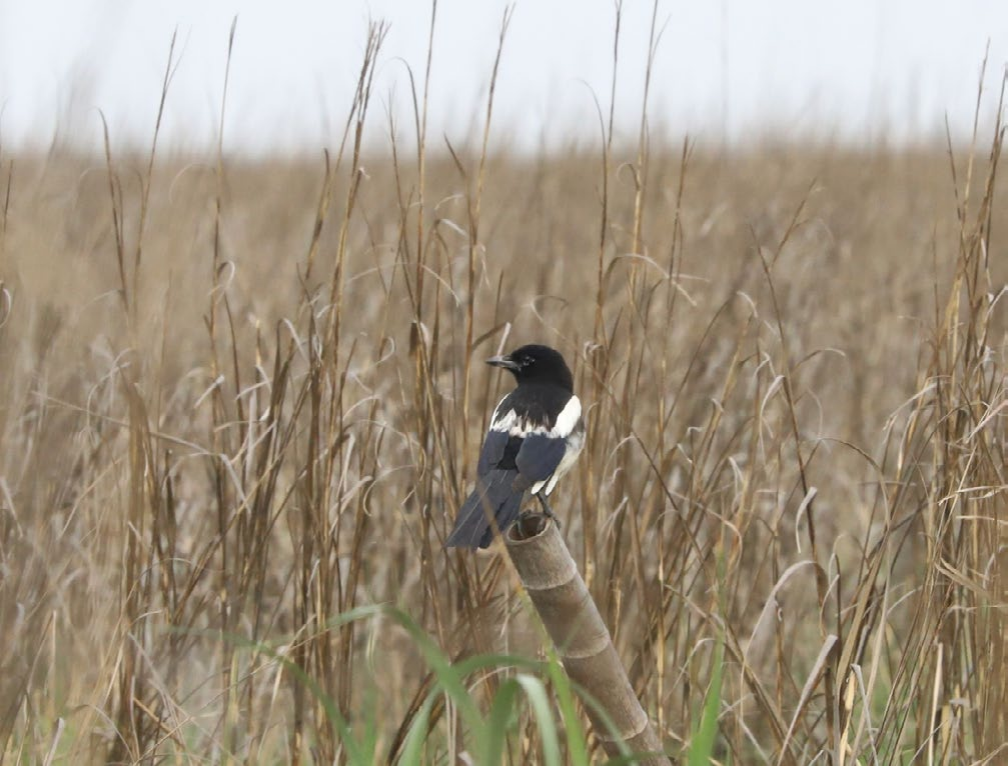
A Eurasian magpie standing in the invasive cordgrass habitat.

Field observations were conducted in the core area of Dafeng Milu National Nature Reserve (32°59′–33°03′ N, 120°47′–120°53′ E), Jiangsu Province, China. The reserve is in the Yellow Sea wetland, China's largest contiguous coastal marsh wetland. The natural coastal landforms of the study area, which features a variety of habitat types and a low level of human disturbance, are preserved, providing suitable habitats for many birds. The native vegetation in the reserve is primarily composed of common reed ( *Phragmites australis* ), cogon grass ( *Imperata cylindrica* ), and weepweed (*Suaeda salsa*). However, due to the long‐term invasion of cordgrass, coastal vegetation has been transformed into a large area of monoculture of cordgrass, while native vegetation has degraded to higher elevations away from the coast (Chen et al. 2019). Therefore, the areas of native bird habitats are decreasing annually, and the number of birds utilizing cordgrass habitats is on the rise, with the vinous‐throated parrotbill serving as the most commonly observed species (Chen et al. 2023). The Eurasian magpie *Pica pica* was also recorded in the cordgrass habitat in our previous survey (Figure 1).

### 2.2 Field Sampling

From May to July 2019, we conducted surveys on the breeding situation of vinous‐throated parrotbill in native vegetation and invasive cordgrass. We first identified currently active vinous‐throated parrotbill territories within the study areas based on previous surveys. Then, we searched for bird nests in each study area from 7:00 to 10:00 AM and again from 3:00 to 6:00 PM on rain‐free days, utilizing behavioral observations of adult birds and systematic search techniques (Conkling et al. 2012; Chen et al. 2020), with a minimum of two observers present for each survey. Once a nest was found, we marked its position using a GPS device (GARMIN, eTrex‐221x) and assessed its status (nesting, incubating, nestling, or inactive) by examining the freshness of the nest material, the contents of the nest, and the behaviors of the parent birds. If a newly discovered empty nest does not contain at least one egg during subsequent observations, it is considered inactive.

We tracked the fate of each active nest (32 nests in native vegetation habitats and 15 nests in invasive cordgrass habitats) through visual inspections every 3 to 5 days and used infrared cameras (Yianws, L710) monitoring to identify predators accurately. Due to variations in the breeding time of different nests, 30 infrared cameras were used in our study, and 17 cameras were recycled for the second nest monitoring after the first nest monitoring. We mounted the cameras on a bamboo pole situated 0.8 to 1.2 m from the bird nest and camouflaged them with the surrounding plant leaves (Herranz, Yanes, and Suárez 2002). Each nest was monitored for approximately 3 to 25 days until failure or success. After a nest predation event, we identified or classified the predator species by examining infrared camera photos and the traces left at the site. For events involving predators that were not documented due to camera malfunctions or other reasons, we infer the type of predator based on the post‐event traces left behind. Specifically, ant predation can be easily verified by the presence of ants covering eggs or nestlings (Twedt et al. 2001), meanwhile, we ruled out the presence of other predators based on infrared camera records. Rats are often associated with eggshell fragments, whereas snakes generally leave no discernible traces after preying on nests (Pietz and Granfors 2000; Klug, Jackrel, and With 2010). It must be acknowledged that this method may lead to incorrect identification. Therefore, we indicated the observations found through this technique in the results. Our work obtained approval from the reserve management office before conducting the experiments.

## 3 Results

**FIGURE 2:**
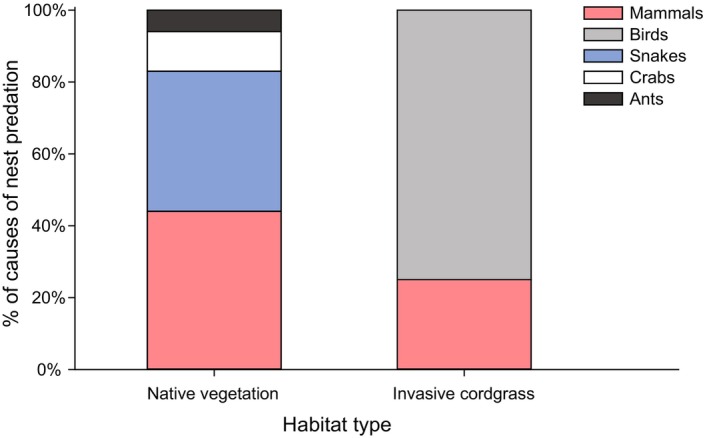
Percentage of different predators of vinous‐throated parrotbill nests in native vegetation (left) and invasive cordgrass habitats (right).

In native vegetation habitats, 18 out of 32 nests were preyed upon (56% of all nests; predators of 14 nests were identified by infrared cameras), two nests (6%) were flooded, seven nests (22%) were abandoned by parent birds, and only five nests (16%) were bred successfully. Eight nests (44% of predated nests) were predated by mammals (five caused by the Siberian weasel *Mustela sibirica* , one nest was identified using nest state; and three caused by the striped field mouse *Apodemus agrarius* ), seven nests (39%) by snakes (all caused by the chifeng ratsnake *Elaphe anomala* , two nests were identified using nest state), two nests (11%) by crabs (all caused by the sesarmid crab *Chiromantes dehaani*), and one nest (6%) by ants (caused by the black ant *Polyrhachis vicina*, identified using nest state) (Figure 2).

**FIGURE 3:**
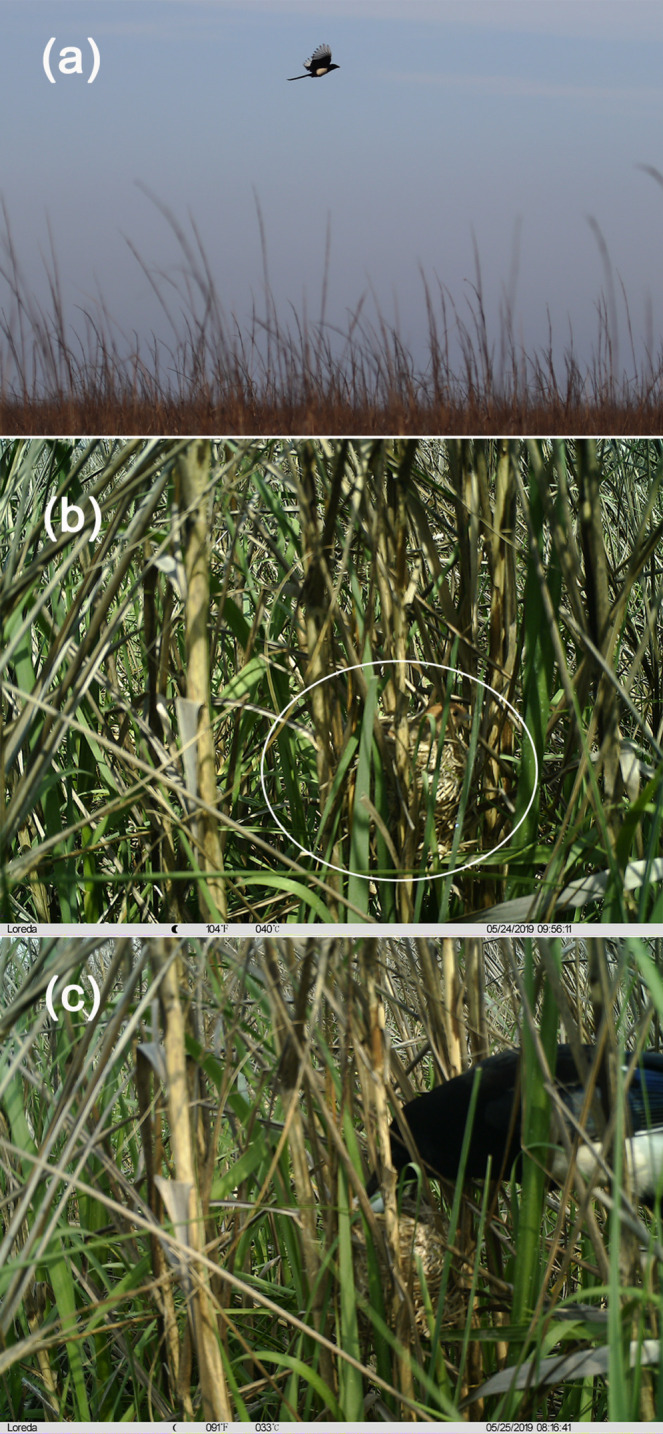
Predation by the Eurasian magpie on the nest of the vinous‐throated parrotbill in invasive cordgrass habitats. (a) magpie searching for prey; (b) adult vinous‐throated parrotbill incubating eggs in the nest; (c) magpie attacking the nest.

In invasive cordgrass habitats, 4 out of 15 nests were preyed upon (27% of all nests; all predators were identified by infrared cameras), six nests (40%) were flooded, two nests (13%) were abandoned by parent birds, and three nests (20%) were bred successfully. One nest (25% of predated nests) was predated by mammals (caused by the striped field mouse), and three nests (75%) were predated by birds (all caused by magpies) (Figure 2). We often observed magpies flying through the sky in cordgrass habitats, and the infrared camera records also demonstrated their adept nest predation skills (Figure 3). Magpies' predation activities were distributed between 6:00 to 12:00 AM and 2:00 to 6:00 PM. They frequently visited the same nest to prey, but did not feed within the nest; instead, they carried the eggs or nestlings away.

## 4 Discussion

Our observations indicate that the main nest predators of parrotbills were mainly mammals and snakes in native habitats, but in cordgrass habitats were birds and occasionally mammals. In many ecosystems, snakes and mammals are likely the most common predators of passerine nests, accounting for more than half of all predation events (DeGregorio et al. 2014; Thompson and Burhans 2003; Weatherhead and Blouin‐Demers 2004). Our findings in native habitats show a similar pattern, with over 80% of nest predations attributable to mammals and snakes. However, the predation rate was quite lower in cordgrass habitats, and the dominant nest predator dramatically changed to birds (magpies), a new predator that has never been found in native habitats. Moreover, no snakes were detected in cordgrass habitats (based on our observation). We speculate that native snakes may not have adapted to cordgrass habitats and that the absence of snake predators reduces the risk of predation on bird nests. Moreover, the overly dense vegetation structure of cordgrass may also limit the search efficiency of ground predators, e.g., mammals (Grant et al. 2006), further explaining the low nest predation rate in cordgrass habitats.

Previous studies based on both artificial or natural nests have shown that magpies are common nest predators across various ecosystems, ranging from urban to rural habitats (Groom 1993; Moser and Ratti 2005; Capstick, Sage, and Madden 2019; Holopainen, Väänänen, and Fox 2020). However, nests preyed upon by magpies typically belong to birds that nest in forest trees, open grasslands, shrublands, and bare ground (Pampush and Anthony 1993; Haegen, Schroeder, and DeGraaf 2002; Dinkins et al. 2012; Skorka et al. 2014), while small passerines that nest in particularly dense vegetation or hedgerows are seldom subject to predation (Capstick 2017). Choosing to nest in areas with greater vegetation cover and higher stem density may be a strategy for birds to evade predators (Salema et al. 2018). The vinous‐throated parrotbill is a typical bird that nests in the understory of dense vegetation in its native habitat (Chen et al. 2023). We frequently encounter magpies in the native habitats of our study area but have never previously recorded them preying on the nests of parrotbills. This may be attributed to the high concealment of parrotbill nests (in the lower part of native vegetation), making it difficult for aerial predators to visually detect their locations. Birds overlapping with magpie breeding areas often experience high nest predation rates (Capstick, Sage, and Madden 2019). However, the invasive cordgrass area is a monoculture and lacks suitable nesting trees for magpies. We speculate that the more easily detectable parrotbill nests attract magpies for predation in cordgrass habitats. To avoid tidal inundation, parrotbills increase their nesting height in cordgrass habitats (the height of the nest above the ground increased from 38.44 ± 2.45 cm to 49.13 ± 3.06 cm, which represents an approximate increase of 29%) (Chen et al. 2023). However, the adaptive behaviors in animals often were two‐sided with both beneficial and harmful consequences (Rodewald, Shustack, and Hitchcock 2010). This change in nesting behavior may increase the exposure of parrotbill nests in cordgrass habitats, especially for aerial predators. Corvids are known for their high intelligence (Emery and Clayton 2004), so magpies may have noticed parrotbills breeding in cordgrass habitats before other native predators and quickly mastered preying skills. In addition, as aerial predators, magpies possess excellent vision and flight ability, which also facilitates locating higher parrotbill nests in monocultured cordgrass habitats more easily than snakes and mammals could.

The impact of cordgrass invasion on coastal ecology in China has received much attention from all sectors, and there has been an increasing number of government‐led coastal restoration projects to eradicate cordgrass and restore bare tidal flats (Lyu et al. 2023). The important goal of these projects is to rescue iconic wetlands and critical shorebird habitats (Ren et al. 2021), and some studies have shown the positive effects of eradicating cordgrass on waterbirds (mainly waders) (Lyu et al. 2023; Ma et al. 2023). However, China's coastal wetlands also host numerous other bird groups, e.g., passerines and raptors, and the impact of long‐term coexistence with invasive cordgrass on these birds is still poorly understood. Studies on the Pacific coast of North America have shown that invasive cordgrass provides important habitats for some birds, e.g., passerines and rails (Lampert et al. 2014; Schwarzer, Cox, and Tornwall 2022), and the thorough eradication of cordgrass even threatens the survival of these species (Lampert et al. 2014). Our previous studies have also indicated that, amidst the increasing degradation of native vegetation habitats, invasive cordgrass could serve as an alternative habitat for some birds (Chen et al. 2022, 2023; Wu et al. 2023). The breeding of native birds in cordgrass habitats may further attract the immigration of native predators. We recommend enhancing the assessment of the status of native birds and other animal groups residing in cordgrass habitats to identify any potential negative effects of cordgrass eradication and the associated uncertain consequences on the entire ecosystem. Therefore, there is an urgent need for multitaxa data based on field surveys for government departments to develop balanced management measures for cordgrass.

Our study suggested that magpies move into invasive cordgrass habitats to prey on parrotbill nests, but interestingly they do not appear to be strongly linked in native habitats. Further long‐term studying and monitoring of predator–prey interactions in invasive cordgrass habitats is needed to assess the potential cascade impact on the trophic web.

## Author Contributions

**Yanhong Chen:** conceptualization (equal), methodology (equal), visualization (equal), writing – original draft (equal), writing – review and editing (equal). **Youle Xu:** data curation (equal), methodology (equal), visualization (equal). **Junjie Wang:** data curation (equal), methodology (equal), visualization (equal). **Taiyu Chen:** investigation (equal), methodology (equal). **Bin Liu:** data curation (equal), investigation (equal). **Pan Chen:** conceptualization (lead), data curation (equal), funding acquisition (lead), investigation (lead), methodology (equal), resources (equal), visualization (equal), writing – original draft (equal), writing – review and editing (lead). **Changhu Lu:** conceptualization (equal), funding acquisition (equal), resources (equal), writing – review and editing (equal).

## Conflicts of Interest

The authors declare no conflicts of interest.

## Data Availability

The raw data used to perform analyses and generate figures are included in the manuscript.

## References

[ece370905-bib-0001] Borgmann, K. L. , and A. D. Rodewald . 2004. “Nest Predation in an Urbanizing Landscape: The Role of Exotic Shrubs.” Ecological Applications 14, no. 6: 1757–1765. 10.1890/03-5129.

[ece370905-bib-0201] Butchart, S. H. M., M. Walpole, B. Collen, et al. 2010. “Global Biodiversity: Indicators of Recent Declines.” Science 328, 1164–1168. 10.1126/science.1187512. 20430971

[ece370905-bib-0002] Capstick, L. 2017. “Variation in the Effect of Corvid Predation on Songbird Populations.” (Doctor of Philosophy) University of Exeter (United Kingdom).

[ece370905-bib-0003] Capstick, L. A. , R. B. Sage , and J. R. Madden . 2019. “Predation of Artificial Nests in UK Farmland by Magpies ( *Pica pica* ): Interacting Environmental, Temporal, and Social Factors Influence a Nest's Risk.” European Journal of Wildlife Research 65, no. 3: 90. 10.1007/s10344-019-1290-6.

[ece370905-bib-0004] Chen, P. , T. Chen , B. Liu , M. Zhang , and C. Lu . 2022. “Song Variation of a Native Songbird in a Modified Habitat by Invasive Plant.” Integrative Zoology 17, no. 1: 93–104. 10.1111/1749-4877.12573. 34216516 PMC9292316

[ece370905-bib-0005] Chen, P. , T. Chen , B. Liu , M. Zhang , C. Lu , and Y. Chen . 2020. “Snakes Are the Principal Nest Predators of the Threatened Reed Parrotbill in a Coastal Wetland of Eastern China.” Global Ecology and Conservation 23: e01055. 10.1016/j.gecco.2020.e01055.

[ece370905-bib-0006] Chen, P. , Y. Chen , H. Chen , et al. 2023. “Vinous‐Throated Parrotbills Breed in Invasive Smooth Cordgrass Habitat: Can Native Birds Avoid the Potential Ecological Trap?” Avian Research 14: 100119. 10.1016/j.avrs.2023.100119.

[ece370905-bib-0007] Chen, P. , Y. Zhang , X. Zhu , and C. Lu . 2019. “Ecological Effects of Invasion by the Smooth Cordgrass *Spartina alterniflora* on Birds (In Chinese).” Acta Ecologica Sinica 36, no. 7: 2282–2290. 10.5846/stxb201801110089.

[ece370905-bib-0008] Clauser, A. J. , and S. B. McRae . 2016. “King Rails ( *Rallus elegans* ) Vary Building Effort and Nest Height in Relation to Water Level.” Waterbirds 39, no. 3: 268–276.

[ece370905-bib-0009] Cockle, K. L. , A. Bodrati , M. Lammertink , E. Bianca Bonaparte , C. Ferreyra , and F. G. Di Sallo . 2016. “Predators of Bird Nests in the Atlantic Forest of Argentina and Paraguay.” Wilson Journal Of Ornithology 128, no. 1: 120–131. 10.1676/wils-128-01-120-131.1.

[ece370905-bib-0010] Conkling, T. J. , T. L. Pope , K. N. Smith , et al. 2012. “Black‐Capped Vireo Nest Predator Assemblage and Predictors for Nest Predation.” Journal of Wildlife Management 76, no. 7: 1401–1411. 10.1002/jwmg.388.

[ece370905-bib-0011] Cook, L. F. , and C. A. Toft . 2005. “Dynamics of Extinction:: Population Decline in the Colonially Nesting Tricolored Blackbird *Agelaius tricolor* .” Bird Conservation International 15, no. 1: 73–88. 10.1017/s0959270905000067.

[ece370905-bib-0012] Cox, W. A. , F. R. Thompson III , and J. Faaborg . 2012. “Landscape Forest Cover and Edge Effects on Songbird Nest Predation Vary by Nest Predator.” Landscape Ecology 27, no. 5: 659–669. 10.1007/s10980-012-9711-x.

[ece370905-bib-0202] Crystal‐Ornelas, R. , and J. L. Lockwood . 2020. “The ‘known unknowns’ of invasive species impact measurement.” Biological Invasions 22, 1513–1525. 10.1007/s10530-020-02200-0.

[ece370905-bib-0203] Davidsdottir, B., T. G. Gunnarsson, G. Halldorsson, B. D. Sigurdsson. 2016. “Avian abundance and communities in areas revegetated with exotic versus native plant species.” Icelandic Agricultural Sciences 29, 21–37. 10.16886/ias.2016.03.

[ece370905-bib-0013] DeGregorio, B. A. , S. J. Chiavacci , P. J. Weatherhead , J. D. Willson , T. J. Benson , and J. H. Sperry . 2014. “Snake Predation on North American Bird Nests: Culprits, Patterns and Future Directions.” Journal of Avian Biology 45, no. 4: 325–333. 10.1111/jav.00364.

[ece370905-bib-0014] Dinkins, J. B. , M. R. Conover , C. P. Kirol , and J. L. Beck . 2012. “Greater Sage‐Grouse ( *Centrocercus urophasianus* ) Select Nest Sites and Brood Sites Away From Avian Predators.” Auk 129, no. 4: 600–610. 10.1525/auk.2012.12009.

[ece370905-bib-0015] Emery, N. J. , and N. S. Clayton . 2004. “The Mentality of Crows: Convergent Evolution of Intelligence in Corvids and Apes.” Science 306, no. 5703: 1903–1907. 10.1126/science.1098410. 15591194

[ece370905-bib-0016] Fisher, R. J. , and S. K. Davis . 2011. “Post‐Fledging Dispersal, Habitat Use, and Survival of Sprague's Pipits: Are Planted Grasslands a Good Substitute for Native?” Biological Conservation 144: 263–271. 10.1016/J.BIOCON.2010.08.024.

[ece370905-bib-0017] Gan, X. J. , C. Choi , Y. Wang , Z. J. Ma , J. K. Chen , and B. Li . 2010. “Alteration of Habitat Structure and Food Resources by Invasive Smooth Cordgrass Affects Habitat Use by Wintering Saltmarsh Birds at Chongming Dongtan, East China.” Auk 127, no. 2: 317–327. 10.1525/auk.2009.09147.

[ece370905-bib-0018] Gao, J. , F. Bai , Y. Yang , S. Gao , Z. Liu , and J. Li . 2012. “Influence of Spartina Colonization on the Supply and Accumulation of Organic Carbon in Tidal Salt Marshes of Northern Jiangsu Province, China.” Journal of Coastal Research 28, no. 2: 486–498. 10.2112/jcoastres-d-11-00062.1.

[ece370905-bib-0019] Gjerdrum, C. , C. S. Elphick , and M. Rubega . 2005. “Nest Site Selection and Nesting Success in Saltmarsh Breeding Sparrows: The Importance of Nest Habitat, Timing, and Study Site Differences.” Condor 107, no. 4: 849–862. 10.1650/7723.1.

[ece370905-bib-0020] Grant, T. A. , E. M. Madden , T. L. Shaffer , P. J. Pietz , G. B. Berkey , and N. J. Kadrmas . 2006. “Nest Survival of Clay‐Colored and Vesper Sparrows in Relation to Woodland Edge in Mixed‐Grass Prairies.” Journal of Wildlife Management 70, no. 3: 691–701. 10.2193/0022-541x(2006)70[691:Nsocav]2.0.Co;2.

[ece370905-bib-0021] Groom, D. W. 1993. “Magpie *Pica pica* Predation on Blackbird *Turdus merula* Nests in Urban Areas.” Bird Study 40: 55–62. 10.1080/00063659309477129.

[ece370905-bib-0022] Haegen, W. M. V. , M. A. Schroeder , and R. M. DeGraaf . 2002. “Predation on Real and Artificial Nests in Shrubsteppe Landscapes Fragmented by Agriculture.” Condor 104, no. 3: 496–506. 10.1650/0010-5422(2002)104[0496:Poraan]2.0.Co;2.

[ece370905-bib-0023] Herranz, J. , M. Yanes , and F. Suárez . 2002. “Does Photo‐Monitoring Affect Nest Predation?” Journal of Field Ornithology 73, no. 1: 97–101. 10.1648/0273-8570-73.1.97.

[ece370905-bib-0024] Holopainen, S. , V.‐M. Väänänen , and A. D. Fox . 2020. “Landscape and Habitat Affect Frequency of Artificial Duck Nest Predation by Native Species, But Not by An Alien Predator.” Basic and Applied Ecology 48: 52–60. 10.1016/j.baae.2020.07.004.

[ece370905-bib-0025] Hunter, E. A. , N. P. Nibbelink , and R. J. Cooper . 2016. “Threat Predictability Influences Seaside Sparrow Nest Site Selection When Facing Trade‐Offs From Predation and Flooding.” Animal Behaviour 120: 135–142. 10.1016/j.anbehav.2016.08.001.

[ece370905-bib-0026] Ibanez‐Alamo, J. D. , R. D. Magrath , J. C. Oteyza , et al. 2015. “Nest Predation Research: Recent Findings and Future Perspectives.” Journal of Ornithology 156: S247–S262. 10.1007/s10336-015-1207-4.

[ece370905-bib-0027] Kamaru, D. N. , T. M. Palmer , C. Riginos , et al. 2024. “Disruption of an Ant‐Plant Mutualism Shapes Interactions Between Lions and Their Primary Prey.” Science 383, no. 6681: 433–438. 10.1126/science.adg1464. 38271503

[ece370905-bib-0028] Klug, P. E. , S. L. Jackrel , and K. A. With . 2010. “Linking Snake Habitat Use to Nest Predation Risk in Grassland Birds: The Dangers of Shrub Cover.” Oecologia 162, no. 3: 803–813. 10.1007/s00442-009-1549-9. 20052494

[ece370905-bib-0029] Lampert, A. , A. Hastings , E. D. Grosholz , S. L. Jardine , and J. N. Sanchirico . 2014. “Optimal Approaches for Balancing Invasive Species Eradication and Endangered Species Management.” Science 344, no. 6187: 1028–1031. 10.1126/science.1250763. 24876497

[ece370905-bib-0030] Li, B. , C. H. Liao , X. D. Zhang , et al. 2009. “ *Spartina alterniflora* Invasions in the Yangtze River Estuary, China: An Overview of Current Status and Ecosystem Effects.” Ecological Engineering 35, no. 4: 511–520. 10.1016/j.ecoleng.2008.05.013.

[ece370905-bib-0031] Lyons, T. P. , J. R. Miller , D. M. Debinski , and D. M. Engle . 2015. “Predator Identity Influences the Effect of Habitat Management on Nest Predation.” Ecological Applications 25, no. 6: 1596–1605. 10.1890/14-1641.1. 26552267

[ece370905-bib-0032] Lyu, C. , S. Zhang , X. Ren , et al. 2023. “The Effect of *Spartina alterniflora* Eradication on Waterbirds and Benthic Organisms.” Restoration Ecology 31, no. 8: e14023. 10.1111/rec.14023.

[ece370905-bib-0033] Ma, Z. J. , C.‐Y. Choi , X. Gan , et al. 2023. “Achievements, Challenges, and Recommendations for Waterbird Conservation in China's Coastal Wetlands.” Avian Research 14: 100123. 10.1016/j.avrs.2023.100123.

[ece370905-bib-0034] Ma, Z. J. , X. J. Gan , C. Choi , and B. Li . 2014. “Effects of Invasive Cordgrass on Presence of Marsh Grassbird in an Area Where It Is Not Native.” Conservation Biology 28, no. 1: 150–158. 10.1111/cobi.12172. 24405105

[ece370905-bib-0035] Moser, A. M. , and J. T. Ratti . 2005. “Value of Riverine Islands to Nongame Birds.” Wildlife Society Bulletin 33, no. 1: 273–284. 10.2193/0091-7648(2005)33[273:Voritn]2.0.Co;2.

[ece370905-bib-0036] Ning, Z. , C. Chen , T. Xie , et al. 2021. “Can the Native Faunal Communities Be Restored From Removal of Invasive Plants in Coastal Ecosystems? A Global Meta‐Analysis.” Global Change Biology 27, no. 19: 4644–4656. 10.1111/gcb.15765. 34170600

[ece370905-bib-0037] Pampush, G. J. , and R. G. Anthony . 1993. “Nest Success, Habitat Utilization and Nest‐Site Selection of Long‐Billed Curlews in the Columbia Basin, Oregon.” Condor 95, no. 4: 957–967. 10.2307/1369431.

[ece370905-bib-0038] Pietz, P. J. , and D. A. Granfors . 2000. “Identifying Predators and Fates of Grassland Passerine Nests Using Miniature Video Cameras.” Journal of Wildlife Management 64, no. 1: 71–87. 10.2307/3802976.

[ece370905-bib-0039] Ren, J. , J. Chen , C. Xu , et al. 2021. “An Invasive Species Erodes the Performance of Coastal Wetland Protected Areas.” Science Advances 7, no. 42: 8943. 10.1126/sciadv.abi8943. PMC8514088 34644105

[ece370905-bib-0040] Robertson, E. P. , and B. J. Olsen . 2015. “Behavioral Plasticity in Nest Building Increases Fecundity in Marsh Birds.” Auk 132, no. 1: 37–45. 10.1642/auk-14-73.1.

[ece370905-bib-0041] Rodewald, A. D. , D. P. Shustack , and L. E. Hitchcock . 2010. “Exotic Shrubs as Ephemeral Ecological Traps for Nesting Birds.” Biological Invasions 12, no. 1: 33–39. 10.1007/s10530-009-9426-3.

[ece370905-bib-0042] Salema, C. A. , G. A. Gale , and S. Bumrungsri . 2018. “Nest‐Site Selection by Common Green Magpie ( *Cissa chinensis* ) in a Tropical Dry Evergreen Forest, Northeast Thailand.” Wilson Journal Of Ornithology 130, no. 1: 256–261.

[ece370905-bib-0043] Schlossberg, S. , and D. I. King . 2010. “Effects of Invasive Woody Plants on Avian Nest Site Selection and Nesting Success in Shrublands.” Animal Conservation 13, no. 3: 286–293. 10.1111/j.1469-1795.2009.00338.x.

[ece370905-bib-0044] Schmidt, K. A. , and C. J. Whelan . 1999. “Effects of Exotic Lonicera and Rhamnus on Songbird Nest Predation.” Conservation Biology 13, no. 6: 1502–1506. 10.1046/j.1523-1739.1999.99050.x.

[ece370905-bib-0045] Schmidt, S. M. , A. M. V. Fournier , J. M. Osborn , and T. J. Benson . 2023. “Water Depth Influences Survival and Predator‐Specific Patterns of Nest Loss in Three Secretive Marsh Bird Species.” Ecology and Evolution 13, no. 12: e10823. 10.1002/ece3.10823. 38089901 PMC10714062

[ece370905-bib-0046] Schwarzer, A. C. , W. A. Cox , and B. Tornwall . 2022. “Habitat Selection of Nesting and Fledgling Salt Marsh Songbirds in Northeast Florida.” Journal of Wildlife Management 86, no. 6: 22260. 10.1002/jwmg.22260.

[ece370905-bib-0047] Skorka, P. , R. Martyka , J. D. Wojcik , and M. Lenda . 2014. “An Invasive Gull Displaces Native Waterbirds to Breeding Habitats More Exposed to Native Predators.” Population Ecology 56, no. 2: 359–374. 10.1007/s10144-013-0429-7.

[ece370905-bib-0048] Smith, D. M. , D. M. Finch , and D. L. Hawksworth . 2009. “Black‐Chinned Hummingbird Nest‐Site Selection and Nest Survival in Response to Fuel Reduction in a Southwestern Riparian Forest.” Condor 111, no. 4: 641–652. 10.1525/cond.2009.090089.

[ece370905-bib-0049] Stewart, P. S. , R. A. Hill , P. A. Stephens , M. J. Whittingham , and W. Dawson . 2021. “Impacts of Invasive Plants on Animal Behaviour.” Ecology Letters 24, no. 4: 891–907. 10.1111/ele.13687. 33524221

[ece370905-bib-0050] Stinson, L. T. , and L. Pejchar . 2018. “The Effects of Introduced Plants on Songbird Reproductive Success.” Biological Invasions 20, no. 6: 1403–1416. 10.1007/s10530-017-1633-8.

[ece370905-bib-0051] Thompson, F. R. , and D. E. Burhans . 2003. “Predation of Songbird Nests Differs by Predator and Between Field and Forest Habitats.” Journal of Wildlife Management 67, no. 2: 408–416.

[ece370905-bib-0052] Twedt, D. J. , R. R. Wilson , J. L. Henne‐Kerr , and R. B. Hamilton . 2001. “Nest Survival of Forest Birds in the Mississippi Alluvial Valley.” Journal of Wildlife Management 65, no. 3: 450–460. 10.2307/3803097.

[ece370905-bib-0053] van Oort, H. , D. J. Green , M. Hepp , and J. M. Cooper . 2015. “Do Fluctuating Water Levels Alter Nest Survivorship in Reservoir Shrubs?” Condor 117, no. 3: 376–385. 10.1650/condor-14-154.1.

[ece370905-bib-0054] Weatherhead, P. J. , and G. Blouin‐Demers . 2004. “Understanding Avian Nest Predation: Why Ornithologists Should Study Snakes.” Journal of Avian Biology 35, no. 3: 185–190. 10.1111/j.0908-8857.2004.03336.x.

[ece370905-bib-0055] Wu, D. , Z. Wang , W. Hu , C. Lu , and P. Chen . 2023. “The Native Reed‐Specific Bird, Reed Parrotbill, Has Been Detected in Exotic Smooth Cordgrass.” Ecology and Evolution 13, no. 8: e10417. 10.1002/ece3.10417. 37575595 PMC10412437

[ece370905-bib-0056] Yilmaz, K. T. , H. Alphan , A. Kosztolanyi , Y. Unlukaplan , and M. A. Derse . 2020. “Coastal Wetland Monitoring and Mapping Along the Turkish Mediterranean: Determining the Impact of Habitat Inundation on Breeding Bird Species.” Journal of Coastal Research 36, no. 5: 961–972. 10.2112/jcoastres-d-19-00091.1.

[ece370905-bib-0057] Zuo, P. , S. H. Zhao , C. A. Liu , C. H. Wang , and Y. B. Liang . 2012. “Distribution of *Spartina Spp*. Along China's Coast.” Ecological Engineering 40: 160–166. 10.1016/j.ecoleng.2011.12.014.

